# Systematic Review of Cerebral Palsy Registries/Surveillance Groups: Relationships between Registry Characteristics and Knowledge Dissemination

**DOI:** 10.4172/2329-9096.1000266

**Published:** 2015-03-23

**Authors:** Donna S Hurley, Theresa Sukal-Moulton, Deborah Gaebler-Spira, Kristin J Krosschell, Larissa Pavone, Akmer Mutlu, Julius PA Dewald, Michael E Msall

**Affiliations:** 1 Department of Physical Therapy and Human Movement Sciences, Northwestern University, Chicago, IL, USA; 2 Functional and Applied Biomechanics Section, Rehabilitation Medicine Department, Clinical Center, National Institutes of Health, Bethesda, MD, USA; 3 The Rehabilitation Institute of Chicago, Chicago, IL, USA; 4 Marianjoy Rehabilitation Hospital, Wheaton, IL, USA; 5 Department of Physiotherapy and Rehabilitation, Faculty of Health Sciences, Hacettepe University, Ankara, Turkey; 6 University of Chicago Comer Children’s Hospital and Kennedy Research Center on Intellectual and Neurodevelopmental Disabilities, Chicago, IL, USA

**Keywords:** Cerebral palsy, Neuromotor disability, Surveillance, Functioning, Enablement

## Abstract

The aims of this study were to provide a comprehensive summary of the body of research disseminated by Cerebral Palsy (CP) registries and surveillance programs from January 2009 through May 2014 in order to describe the influence their results have on our overall understanding of CP. Secondly, registries/surveillance programs and the work they produced were evaluated and grouped using standardized definitions and classification systems.

**Method:**

A systematic review search in PubMed, CINAH and Embase for original articles published from 1 January 2009 to 20 May 2014 originating from or supported by population based CP registries and surveillance programs or population based national registries including CP were included. Articles were grouped by 2009 World CP Registry Congress aim, registry/surveillance program classification, geographical region, and the International Classification of Function, Disability and Health (ICF) domain. Registry variables were assessed using the ICF-CY classification.

**Results:**

Literature searches returned 177 articles meeting inclusion criteria. The majority (69%) of registry/surveillance program productivity was related to contributions as a Resource for CP Research. Prevention (23%) and Surveillance (22%) articles were other areas of achievement, but fewer articles were published in the areas of Planning (17%) and Raising the Profile of CP (2%). There was a range of registry/surveillance program classifications contributing to this productivity, and representation from multiple areas of the globe, although most of the articles originated in Europe, Australia, and Canada. The domains of the ICF that were primarily covered included body structures and function at the early stages of life. Encouragingly, a variety of CP registry/surveillance program initiatives included additional ICF domains of participation and environmental and personal factors.

**Interpretation:**

CP registries and surveillance programs, including novel non-traditional ones, have significantly contributed to the understanding of how CP affects individuals, families and society. Moving forward, the global CP registry/surveillance program community should continue to strive for uniformity in CP definitions, variables collected and consistency with international initiatives like the ICF so that databases can be consolidated for research use. Adaptation to new technologies can improve access, reduce cost and facilitate information transfer between registrants, researchers and registries/surveillance programs. Finally, increased efforts in documenting variables of individuals with CP into adulthood should be made in order to expand our understanding of CP across the lifespan.

## Introduction

Cerebral palsy (CP) is a neuromuscular disorder caused by an injury to the fetal or infant brain that affects the development of movement and posture and causes activity limitations. The motor disorders of CP are often accompanied by disturbances of sensation, perception, cognition, communication, and behavior; by epilepsy; and by secondary musculoskeletal problems [1]. This consensus definition not only acknowledges the initial static or non-progressive injury to the immature brain but also recognizes the resulting dynamic and evolving medical, developmental, and social issues that this disruption in normal brain development creates throughout the life span [2,3]. Despite advances in technology, pre and postnatal care and identification of risk factors, reported prevalence of CP remains at an average of 2.11 per 1,000 live births across different areas of the globe [4].

To understand the complexity of cerebral palsy, registries using total population of a geographically defined area as the denominator were established and became instrumental in the contribution to our understanding about prevalence [5–7], risk factors [8], etiology [9], and perinatal care [10,11]. The first population-based CP registries were started in Denmark (1950) [12,13], Sweden (1954) [14], England (1966) [15], and Ireland (1966) [16]. Western Australia followed in 1975, with 1956 as its first birth year cohort [17]. A large part of the success of these early registries was related to the ability to capture all children with a diagnosis of CP within a geographically defined region using government programs as resources and referrals.

Over time, the quest to understand CP, its prevalence and prevention grew globally. Funding mechanisms, government healthcare, and social service programs differ between and within countries, necessitating innovative ways to collect data and perform population-based CP research [18–23]. New ascertainment methods for such studies included CP data gathered through multi-source methods, use of government registries and census type surveillance methods to obtain population information.

At the 3rd International Cerebral Palsy Conference (Sydney, Australia, 2009), the Research Foundation of the Cerebral Palsy Alliance hosted a World CP Register Congress, providing an opportunity for global CP registries and surveillance programs to meet and discuss practices and strategies [24]. This Congress facilitated the sharing of information and discussion of issues affecting CP registries, including but not limited to: variables collected, data sources, methods of ascertainment, enrollment, inclusion and exclusion criteria and registry/surveillance program aims.

Although the scientific community has seen a diversification in structure and characteristics of CP registries/surveillance programs over the past decades, there have also been international initiatives to standardize language used to define and classify CP and frameworks to conceptualize health conditions and disorders. One such initiative to unify domains in which clinicians and researchers think about health status in a broad sense was the 2001 World Health Organization’s (WHO) International Classification of Functioning, Disability and Health (ICF). This model acknowledges the body structure and function aspects of a condition, but also examines how it impacts activities of daily life, participation within society and influence of environmental and personal factors [25].

The purpose of this systematic review was to examine and evaluate the research productivity of international CP registries/surveillance programs since the 2009 World CP Register Congress, as it relates to registry characteristics. Specifically, articles were evaluated using established CP registry and surveillance programs’ aims, the type of registry/surveillance program that produced the data and region where it is located. The ICF domains represented in the articles as well as in database variables of known CP registries/surveillance programs were also examined.

## Methods

### Search strategy

A systematic search was conducted by one author (DSH) in collaboration with a medical librarian in MEDLINE (PubMed), Embase, (embase.com) and CINAHL (EBSCO) for original articles published in peer reviewed journals between January 1, 2009 and May 20, 2014. The year of the World CP Register Congress was chosen as a starting date because this is when aims were defined. Controlled vocabulary terms specific to each database were used, based on the terms profiled in the Congress report [24], including cerebral palsy, registry, register, population-based study, and known CP registries and regions. Other articles found incidentally in the process of screening abstracts that fit the criteria were also included.

### Article selection

Inclusion criteria for this systematic review were as follows: (1) full length research articles; (2) written in English; (3) published January 2009 – May 20, 2014; (4) pertaining to cerebral palsy either as a primary patient population or as a primary outcome or endpoint; and (5) participant recruitment or data extracted from a population-based database from a defined region.

Exclusion criteria for this study were as follows: (1) conference abstracts/presentations; (2) editorials, letters to the editor; (3) CP not a primary aim or endpoint of the study; (4) databases that were limited to non-total population such as individual hospitals, clinics, private insurance companies; (5) recruitment from only the general population, convenience and population cohort groups; (6) articles describing future studies (protocols); (7) non-English articles; (8) systematic reviews, Cochrane systematic reviews and meta-analysis papers.

**Figure 1:**
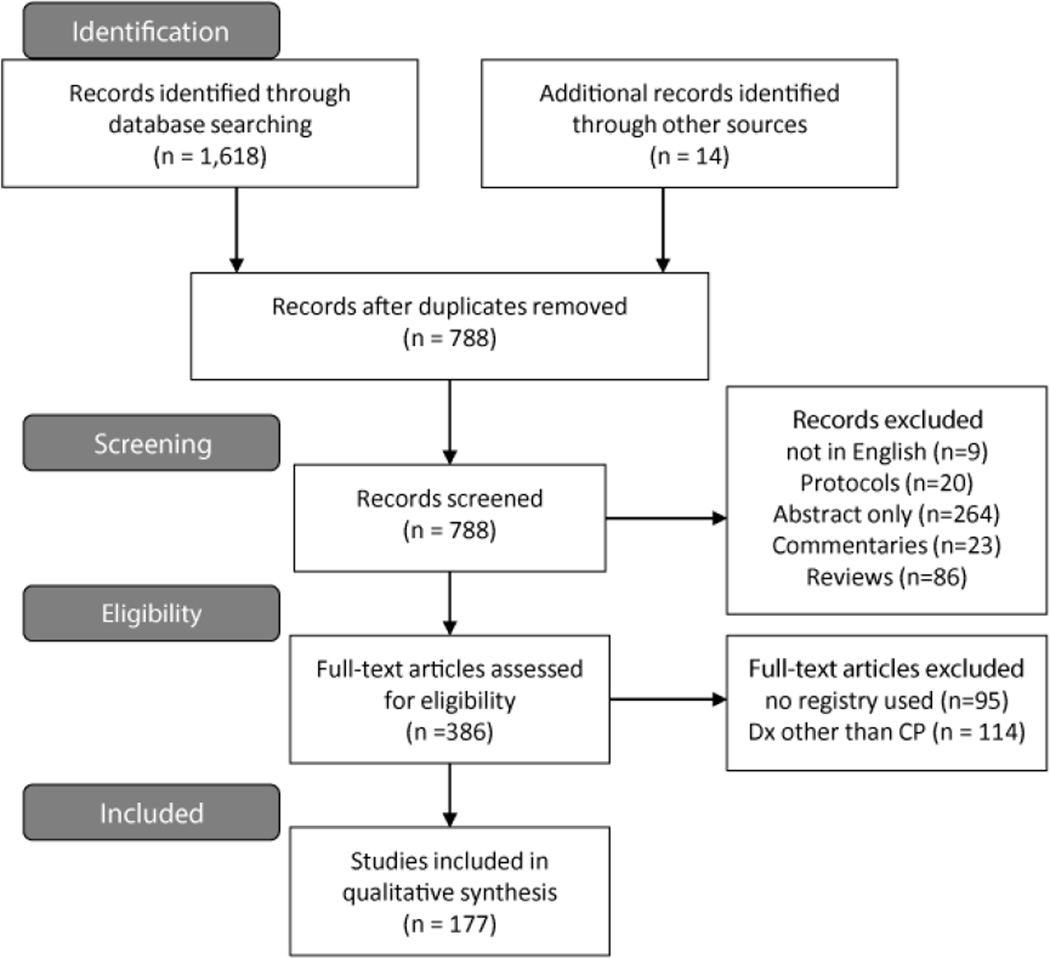
PRISMA diagram of articles reviewed.

The search resulted in 1,618 abstracts with an additional 14 articles found through incidental discovery (for example, abstracts that were identified as part of a conference proceeding, but resulted in a journal article). All Cochrane systematic reviews, systematic reviews and meta-analyses (n=42) were reviewed by three authors (MEM, DGS, LP) for additional articles (n=1) that had potential to fit inclusion criteria. Duplicates were removed and the remaining 788 abstracts were screened for inclusion. These were reviewed independently by two authors (DSH, TSM) using a data abstraction criteria sheet that was comprised of inclusion/exclusion criteria. Abstracts were retained for full review if they met the inclusion criteria or if more information was needed from the full text to ascertain inclusion. A summary PRISMA flow chart [26,27] of the articles identified can be found in Figure 1.

From the 788-screened abstracts, 177 met the inclusion criteria for this study. Using decision guidelines, two independent reviewers (DSH, TSM) categorized each article by primary aim(s), type of CP registry/surveillance program as classified by the CP registry/surveillance program classification system, geographical region captured, and ICF domains represented. Discrepancies were discussed and a final decision was made without requiring an additional independent reviewer. Further details on these categorizations are in the following sections.

### Aims of CP registries/surveillance programs

Consensus was reached at the 2009 World CP Register Congress24 regarding aims and purposes that CP registries should aspire towards. In summary, the CP Registry and Surveillance Program Aims24 include:

#### Planning

This aim asserts that CP registries/surveillance programs, through their research data, can assist families and professionals with the development and planning of medical, social and educational services.

#### Prevention

CP registries/surveillance programs have a unique position to assist with determining etiological understanding and prevention by using the framework of multiple causal pathways to CP.

#### Raise the profile of cerebral palsy

This aim focuses on the potential for CP registries/surveillance programs to increase awareness of CP among community and professional groups through publications, advocacy and social media.

#### Resource for cerebral palsy research

This aim asserts that CP registries/surveillance programs are able to use their registered cases as a source of subjects for etiological or management research in several ways. Firstly, they can be used to investigate the generalizability of research results generated from more limited samples of persons with CP. Secondly, databases can be used as a means of identifying CP as an outcome in long term follow up studies. Thirdly, registry observations can be used as a source of hypothesis-generating preliminary evidence concerning causal pathways or management approaches. When these hypotheses are further tested, registered cases can form a sampling frame.

#### Surveillance

CP registries/surveillance programs can be used to monitor trends over time and determine prevalence of the diagnosis within a defined population.

Two additional uses for CP registries/surveillance programs emerged during abstract screening that were not identified in the original 2009 CP registry aims description. These were CP registries have been used as (1) a recruitment source for independent studies and (2) as a cross-validation tool to confirm or identify the diagnosis of CP within a different sample set. We included these additional purposes within the Resource for CP Research aim.

Journal articles were assigned either one or two aims, depending on the use of CP registry/surveillance program data and stated purpose or outcomes of the study. Those with one aim aligned closely to just one of the stated aims above. Instances where two aims were assigned included prevention and surveillance studies that used CP registry/surveillance program enrollees’ clinical data as a resource or when a study’s conclusions could be used for planning or raising the profile of CP. It also occurred when the paper reported an independent, non-CP registry/surveillance program study that used a CP registry/surveillance program for subject recruitment, confirmation of a CP diagnosis or to link data with government datasets.

### CP registry/surveillance program classification system

In addition to long-established CP registry/surveillance programs, additional avenues researchers used to obtain population data were identified. Therefore, CP registries/surveillance programs were classified in a new, novel way using dictionary definitions and registry/surveillance program descriptions referred to in the literature.

#### Traditional CP registries

These are registries that maintain databases using the total population of a specific geographical region as their denominator, and are often able to link enrollees through government agencies. Examples include the Victorian CP registry and the Danish National CP Registry [28–30].

#### Collaborative CP registries

Traditional CP registries from multiple regions merging data to establish a collaborative database using common language and variables in order to expand CP research efforts. Examples include the Surveillance of CP Europe (SCPE) and the Australian CP Register (ACPR) [31,32].

#### Collaborative CP registry subgroup

Collaborative CP registry subgroups leverage existing collaborative CP registry data and enrollees for research studies, often adding ICF domains not commonly found in traditional CP registries (activity, participation, environment and personal factors). An example is the Study of Participation of Children with Cerebral Palsy Living in Europe (SPARCLE) [33].

#### CP surveillance programs

CP Surveillance programs use methodical, multi-source data collection of variables for a regional CP population when government registry data is unavailable or unattainable. An example includes the Metropolitan Atlanta Developmental Disabilities Surveillance Program (MADDSP) [23].

#### National health surveillance programs for CP

Health care programs have been established for children with CP in certain geographic regions. These surveillance programs aim to prevent hip dislocation, scoliosis and severe contracture deformities and collect and store longitudinal data on children with CP in their databases. Examples include CP Uppföljnings Program (CPUP) in Sweden [28] and CP Oppfølgings Program (CPOP) in Norway [34,35].

#### Government registries and programs

Government sponsored total population datasets with indicators of CP and other related neurodevelopmental disabilities can be used independently or linked with CP registries/surveillance programs for research purposes. Examples include the use of Hospital and Patient Registers and the California Department of Developmental Services [36,37].

#### Census and local CP surveillance programs

These types of programs obtain a total population denominator for CP prevalence in a targeted geographical area through various methods such as door-to-door interviews and population surveys. Examples include prevalence studies in defined areas of Egypt, Pakistan and India [19,21,22].

### Regions of the world of identified articles

Regions were divided into six global areas based on geographical boundaries: Africa, Asia, Australia, Canada, Europe and the United States.

### ICF domains of identified articles

One or more ICF domains were identified and categorized for each article according to the World Health Organization International Classification of Functioning, Disability and Health (ICF) definitions for health condition, body structure and function, activities, participation, environmental factors, and personal factors [25,38].

Of note, articles focusing primarily on surveillance and identification of CP as a diagnosis were categorized as health condition, and those focusing on risk factors related to prenatal influences (in vitro fertilization status or pre-eclampsia for example) were identified as environmental factors because these issues were external to the child with the CP diagnosis.

### CP registry data elements and the ICF-CY

In order to put the productivity of CP registries related to the ICF domains into context, we also compared database variables of 14 global CP registries that were compiled for the 2009 World CP Register Congress with the ICF for Children and Youth (ICF-CY) developmental code sets published by Ellingsen and Simeonsson [39,40]. The code sets were developed through the Delphi technique [41] of formalized consensus among experts, and conceptualize the ICF model into essential categories of a child’s function over 4 distinct developmental periods. They are not restricted to CP, but were chosen to evaluate registry variables because they demonstrate a holistic representation of health and function as it relates to the growing child, which is relevant with the newest definitions of cerebral palsy.

For each of the registries available (Australian CP Register, New South Wales and ACT CP Register, Victorian CP Register, Western Australia CP Register, Surveillance of CP in Europe, Registre des Handicaps de l’Enfant et Observatoire Périnatal de l’Isère et des deux Savoies, Norwegian CP Register, CP Register of Western Sweden, CPUP Sweden, Mersey and Cheshire CP Register, North of England Collaborative CP Register, Autism and Developmental Disabilities Monitoring Network, Metropolitan Atlanta Developmental Disabilities Surveillance Program, and CP Research Registry), we compared their database variables 24 with the ICF-CY code sets [39,40]. We identified if all (14 registries), some (between 1 and 13 registries, inclusive), or none (0 registries) collected variables that related to the code sets. This process was completed for each domain of the ICF-CY and across 4 different age bands (0–2 years, 3–5 years, 6–12 years, and 13–17 years of age). International standards of when a child can be diagnosed with CP, the range is usually between 3–5 years of age. However, certain types of CP, particularly hemiplegia, can be diagnosed at an earlier age, therefore the age band of 0–2 years has been included in this paper.

## Data Analysis

Descriptive statistics were compiled for the regions of the world, registry/surveillance program classification, ICF variables, and aims. Further, themes were identified among research studies within each of the established registry/surveillance program aims.

## Results

In this systematic review, we evaluated the productivity of global CP registries/surveillance programs through peer-reviewed publications on a number of features, including registry/surveillance program aims addressed, registry/surveillance program classification, global region, and ICF domains covered. A total of 177 articles met the criteria for this review (Figure 1 and Tables S1–S11). CP registry/surveillance program data elements capture the highest percentage of variables in the ICF-CY body structure and functions domain, and in the early years of a participant’s life. However, the overall productivity of CP registries/surveillance programs demonstrates consistent output of new evidence across multiple aims and themes with data collection from across the globe and with a strong representation of ICF domains.

### Article characteristics

#### Productivity, regions and registry/surveillance program classification in publications

**Figure 2:**
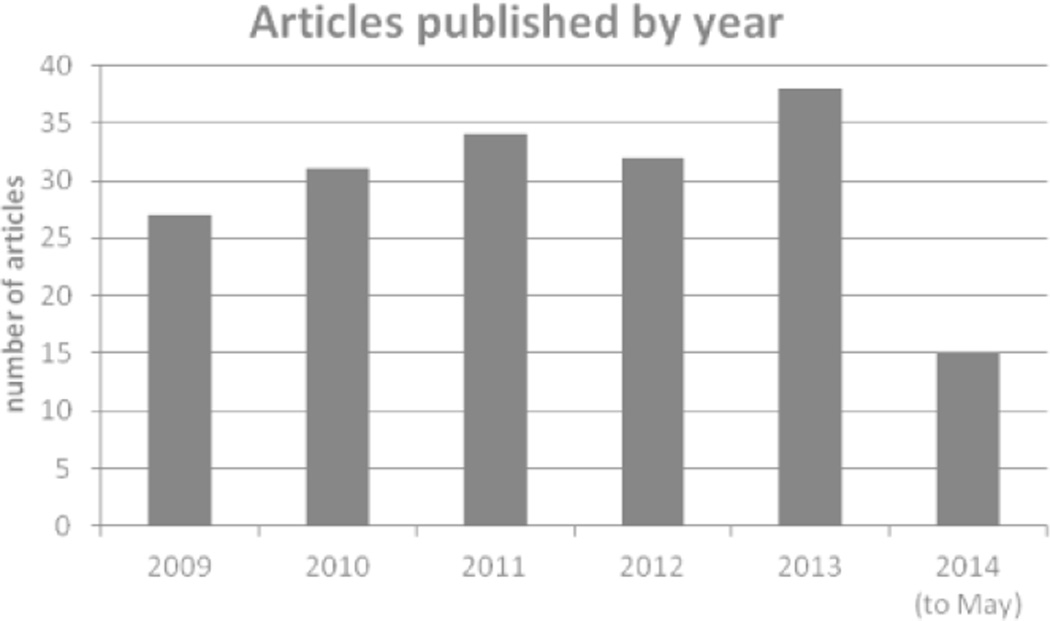
Articles that met inclusion criteria each year. The number of articles identified has increased each full year since 2009.

CP registries/surveillance programs have shown consistent productivity in the years reviewed and are trending upwards, with 27 articles in 2009, increasing to 38 articles in 2013 (Figure 2). The regions that have published the most CP articles are skewed relative to the world’s population (Table 1 and Figure S1). The highest number of articles came from Europe, with more than half of all articles identified originating in this region (n=101), followed by Australia (n=44) and Canada (n=16). Between these three regions, there were 11 traditional CP registries, 4 CP registry collaborative groups, 5 National Health Surveillance programs and 16 government registries or programs (Table S12) identified in our literature review, indicating strong database infrastructures and research support.

In contrast, the articles identified as coming from the Unites States (n=11), Asia (n=4) and Africa (n=1) were significantly fewer in numbers. These regions do not have traditional CP registries in place and instead must rely on surveillance programs, government databases and census methods to ascertain the impact of CP within their countries and regions. No articles were identified in Central or South America or the Middle East.

Research productivity showed an unexpected distribution within the registry classification system. Although the registry classification most frequently responsible for production of research articles were traditional CP registries (n=87), there has been substantial productivity from other types of registries as well. More than half of the identified articles came from a combination of other sources, including collaborative registry databases (n=11) and their subgroups (n=14), surveillance programs (n=7), government CP health programs (n=17), government registries and programs (n=35) and population-based surveillance through local surveillance and census techniques (n=6) (Table 1). Most regions had less than 3 registry classifications found in the papers published. Europe was the only region where every registry classification was represented by at least one article; with the advantage of 26 total registries/surveillance programs identified originating in this region (Table S13).

#### Registry/surveillance program aims in publications

**Figure 3:**
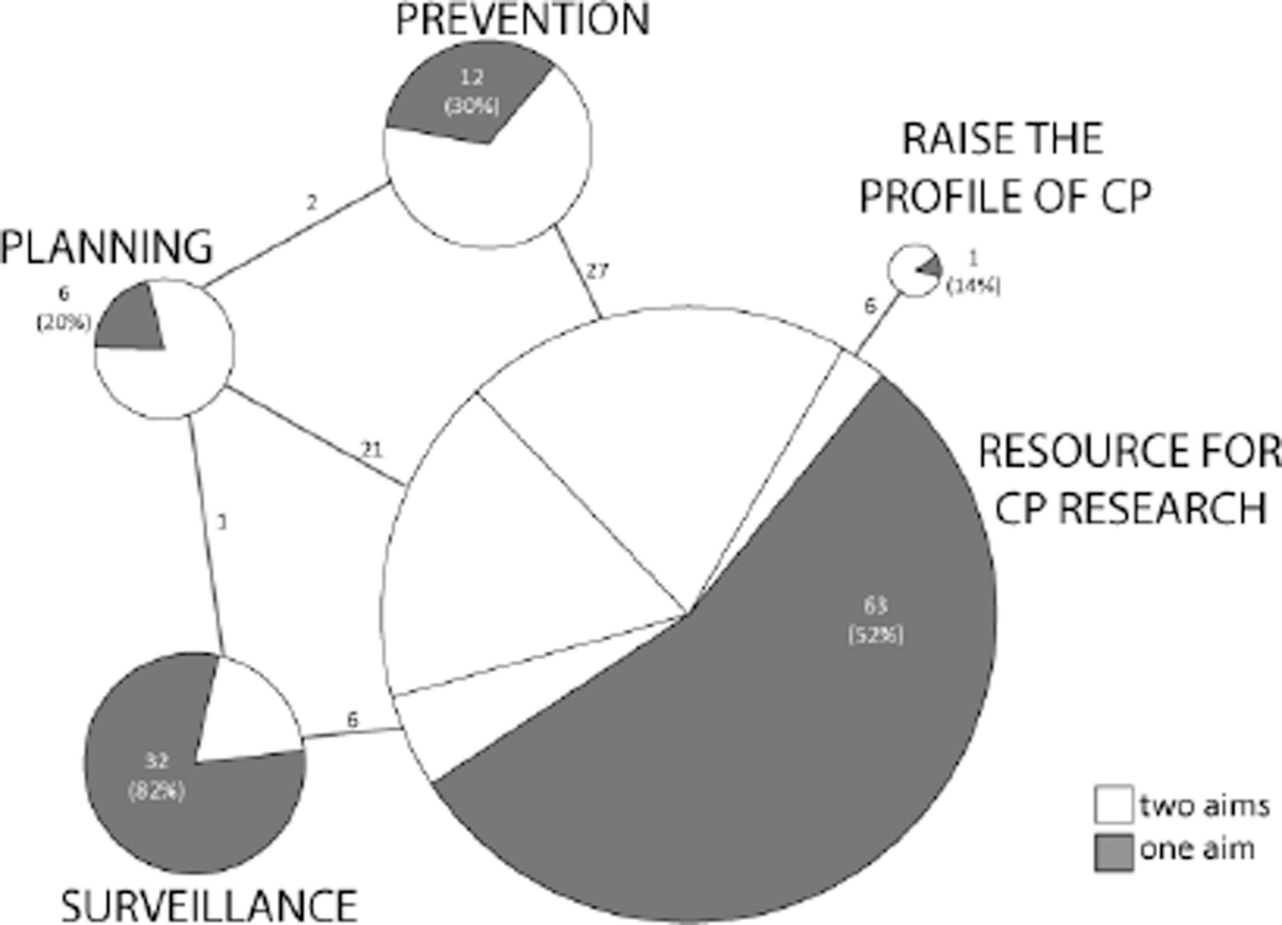
CP Registry aims addressed. This figure summarizes how the 177 articles were categorized according to aim. The size of the pie chart representing each of the 5 aims is scaled to the number of articles in that aim. Within each aim, the pie chart is split between articles that addressed only one aim (dark gray, with number of articles assigned and percent of that aim) and those that addressed 2 (white, number of articles shown on connecting lines). When article(s) addressed two aims, it is shown by a line connecting the pie chart of each aim. For example, 6 articles were classified as addressing both surveillance and resource for CP research.

Article aims were categorized using the CP Registry and Surveillance Groups aims described previously. Of the 177 identified articles, 114 addressed one aim, and 63 accomplished two aims. These results and the linkages between aims are summarized in Figure 3. The aim with the highest number articles was Resource for CP Research (n=123), followed by Prevention (n=41), Surveillance (n=39), Planning (n=30) and Raising the Profile of CP (n=7). Of the articles classified as accomplishing two aims, 60 (95%) fulfilled Resource for CP Research as one of its aims. Three remaining articles that had two aims were categorized as Planning + Prevention (n=2) and Planning + Surveillance (n=1).

Resource for CP Research was identified as a single aim in 63 studies (Table S1), covering a wide range of topics. Themes that emerged included comorbidities of CP [42–59]. CP assessment tools [60–69], issues related to quality of life, pain, school function and stress [70–79] and motor function [80–90]. Other studies focused on the use of MRI and imaging [91–95], genetics [96,97], intervention [98–100], CP registry issues [101,102], prediction of function [103] and subject matter relevant to CP including goal writing and attainment [104].

The remaining 60 studies in this aim were classified as two-aim studies because their central theme fulfilled a different aim, but the way the CP registry/surveillance program was used fit into the Resource for CP Research aim. In these two-aim articles, researchers used a CP registry/surveillance program for study subject recruitment, analysis of the CP registry/surveillance program data, linkage of CP registry/surveillance program data with one or more government registries, or to confirm or identify a CP diagnosis for cases found in government registries data. Central themes of these two-aim articles are discussed in the following, respective sections.

The Prevention aim had 41 articles, 29 of which addressed two aims. The 12 articles addressing Prevention alone (Table S4) including content on maternal risk factors [105–108], infant infection [109–111], reproductive technology [30,112], preterm [113] and term [114] risk factors and antenatal factors associated with perinatal stroke [18].

Twenty-seven of the 29 dual-aim articles were shared with Resource for CP Research (Table S6). Risk factors and casual pathways for CP was the most prevalent theme for this aim, and included investigation of maternal risk factors [115–120], genetics [121–124], term birth [125–128] and labor and delivery [37,129,130]. Other topics of research included preterm risk factors [131,132], reproductive technology [133,134], multiples [135,136], infant infection, [137] MRI findings and risk factors [138] ethnic/socioeconomic disparities [139] and predicting outcomes [140]. The two other Prevention studies related to predicting outcomes were classified concurrently with the Planning aim (Table S5) [141,142].

Prevention is a complex issue to address, and there was evidence of a multi-faceted approached to information gathering in the studies reviewed, with extensive linking of multiple sources to obtain study data. Cross-referencing databases increases the validity and confidence of research studies. Most studies used either a traditional CP registry (n=25) or government registry and program dataset(s) (n=14) as one of the sources. Examples of additional data sources include hospital/physician medical records, birth/death registries and insurance databases, with some papers using as many as 4 different resources to compile study data [143].

The Surveillance aim had 39 articles, 32 of which were focused on this aim alone (Table S2). Prevalence of CP within a defined region(s) was the primary focus of the 32 one-aim Surveillance articles. Eleven articles reported CP prevalence within specific birth years [28,144–153] 6 studies were the first to report CP prevalence data within their respective countries [19,21,22,154–156] and 3 studies reported on regional prevalence [157–159]. Other articles addressed prevalence of comorbidities [160–166], CP registry logistics [167,168], and prevalence of term/post natal [169,170] and preterm births [171]. Surprisingly, traditional CP registries accounted for a relatively small percentage of the prevalence articles retrieved from the search (n=9). Government registries and programs (n=7), collaborative CP registries (n=5) closely followed, with census and local CP surveillance groups (n=5), CP surveillance programs (n=4) and national health surveillance programs for CP (n=1) accounting for the remainder (Table S13). The distribution of varied registry classifications used in surveillance of CP, especially government datasets and census surveillance techniques, demonstrates that alternative ways to gather population based data for CP research can compliment traditional CP registry approaches.

The 7 Surveillance articles that addressed two aims were generated from traditional CP registries (n=3), collaborative CP registries (n=2), national health surveillance programs for CP (n=1), and CP surveillance programs (n=1) (Table S13). Six of the articles were associated with the Resource for CP Research aim (Table S3) with topics including racial and socio-economic disparities [172,173], motor function [174,175], congenital anomalies [176], and prevalence of dyskinetic CP [177]. The remaining article linked with the Planning aim (Table S9), surveying survival rates into adolescence [178].

The 30 articles in the Planning aim addressed planning for current and future needs of children with CP and their families, and how communities can promote social and environmental changes for the success of this group. Data for the Planning articles were obtained from traditional CP registries (n=11), government registries and programs (n=4), national health surveillance programs for CP (n=6), CP surveillance programs (n=5), collaborative CP registries subgroups (n=3), and collaborative CP registries (n=1) (Table S13).

Single aim Planning articles (Table S7) emphasized financial planning [179–181], motor and mental outcomes [182,183] and economic achievements of persons with CP [184]. Life expectancy for children with CP without severe impairments is marginally less than those without CP, therefore planning for future health, educational, environmental and social needs is a necessity, and one that can increase a person’s ability to be an independent and productive member of society [185].

Twenty-one dual aim articles shared this aim (Planning) with the Resource for CP Research aim (Table S8) and emphasized a variety of health [186–191] and hip issues [192–195], planning for environmental and social needs [196–199] equipment [200–202] financial planning [203,204] predicting outcome [205] and mother’s health [206]. Additional two-aim articles have been previously discussed under Prevention (n=2) and Surveillance (n=1).

Only 7 articles in this review were classified as Raising the Profile of CP. The single-aim article in this aim (Table S11) described the successful collaboration between government registries and consumers regarding consensus on whether notification to the registers should be statutory or only with consent [207], demonstrating a critical consultation with registrants. Six of the 7 articles had dual aims with Resource for CP Research (Table S10), focusing mainly on hip surveillance programs [208–211], but also the development of an online national CP Research Registry3 and an article calling for concerted actions by professionals to influence legislation and policy to address environmental access and support services for those with CP [212]. With the paucity of articles for this aim, it could be proposed that traditional peer-reviewed journals may not be the best avenue for articles promoting CP advocacy and awareness. Social media and Internet sites, with their ability to send information globally to millions of people instantly might be a more productive outlet for this aim.

#### ICF domains in CP registry/surveillance programs in publications

Table 2 shows the ICF domains covered in the reviewed articles, in relationship to the CP registry/surveillance program classification. Information from the different ICF domains give a more complete understanding of the whole person, contributing insights about the impact CP has on a person’s health, life activities and their engagement in the community. Many articles addressed more than one ICF domain. The body structure and function domain is the most frequently occurring, and there is a relative lack of evidence being generated in the areas of participation and environmental factors outside of prenatal considerations. We found that articles published in later years of our sampling frame tended to capture more than one area of the ICF, with 9 articles in 2009 and 14 in 2013. This may demonstrate an increased awareness of the inter-connected nature of these domains and the risk of drawing inappropriate conclusions if one domain is examined in isolation.

#### ICF-CY codes and CP registry/surveillance program variables in publications

Data elements from 14 registries were reported in the 2009 World CP Register Congress survey [24], representing approximately one third of the registries indentified during this review. Of those where collected variables were available, 8 were traditional CP registries, 1 was a national health surveillance programs for CP, 2 were collaborative CP registries, 2 were surveillance programs, and 1 was from a local surveillance group. The distribution of registry variables that fulfill the ICF-CY code sets [39,40] for each age band is shown in Table 3. It is notable that a high percentage of the ICF-CY codes in the body structure and function domain are collected by at least some registries/surveillance programs in the early years of a life for a child with CP, but this decreases to less than half of the available codes in this domain by age 6. Percentage of variables in the activities and participation and environmental factors code sets that are represented by at least some registries/surveillance programs fall below 50% by age 3. Given that most of the profiled CP registries/surveillance programs were started before the acceptance of the ICF model, this is not a surprising finding, but one that should be noted when comparing newer registries/surveillance programs to long-established ones. There are several variables that were common to all registries/surveillance programs surveyed. They included: date of birth, gender, mothers’ date of birth, birth weight, gestation, number of fetuses, diagnosis/motor type, epilepsy/seizures, Gross Motor Function Classification System, intellectual function, and post-neonatal cause/timing [24].

## Discussion

This systematic review provides a comprehensive summary of research disseminated by cerebral palsy registries and surveillance programs, and demonstrates the influence these programs have on our overall understanding of Cerebral Palsy (CP) through research themes and within the ICF framework. CP registries/surveillance programs were characterized by their, region, classification, and when known, how their data elements fulfilled ICF-CY developmental code sets.

Establishment of the first CP registries/surveillance programs was done with strict regard to epidemiological considerations. They interfaced with supportive government health care infrastructure to fully ascertain a total CP population within their defined region, and used this cohort as their denominator [213]. This definition of a traditional CP registry has been the gold standard for several decades; however, current global healthcare and funding mechanisms makes this model not achievable across all global regions. This review identified and defined a novel classification system of CP registries/surveillance programs that have been used to garner population-based information regarding CP. They are regional collaborative CP registries, collaborative CP registry subgroups, CP surveillance programs, National health surveillance programs for CP, government sponsored registries and programs and census and local CP surveillance groups. Our acknowledgement of all 7 CP registry types has resulted in a comprehensive evaluation of population-based research that has been disseminated through journal articles.

There are many areas of the globe that are grossly under-represented in population-based studies of CP. As international research productivity expands, it is unknown if the findings of the studies presented in this review can be appropriately applied to the under-represented regions that have different genetic makeup and cultural values. Developing CP registries/surveillance programs in these uncharted regions and then comparing data with established registries/surveillance programs would be a highly effective way of accelerating discovery of the role of genotype and environment.

While there remain strengths and weaknesses with each CP registry/surveillance program classification that are outside of the scope of this review, it is significant to note that research in the field is no longer dependent on any one type of registry/surveillance program, and there may be room for additional strategies in the future as well. Agreement on common language, establishing common data elements for CP [214–216] and rigor in data obtainment and verification must be maintained across registries/surveillance programs to uphold the standards that have been set by traditional CP registries.

The standardized collection of investigational data is a method for facilitation of data captures, comparison of results across studies and aggregation of information into significant metadata results. This effort has been undertaken in other neurological disorders through a process facilitated by the National Institute of Neurological Disorders and Stroke [216] in the United States and significant efforts have been made recently in developing a core data set of ICF-CY code sets specifically for CP [217]. Although not available in time to be implemented in this systematic review process, these two efforts have the potential to help guide new and established registries. Importantly, core datasets need to be carefully planned and developed to maximize the amount of data collected while minimizing burden on the registrant [3].

The advantage of adopting international initiatives such as the ICF is that they provide the opportunity to achieve a comprehensive and holistic understanding of individuals with CP. With the ICF-CY, it was found that less than half of the relevant codes were collected after early childhood. This decline in variables by age reflects poorly on how CP registries/surveillance programs are equipped to research adolescent and adult issues. Variables that capture information on aging with CP, such as medical and developmental associated conditions, quality of life issues and education, employment and housing opportunities should be incorporated into data collection. Similarly, the use of the ICF in research articles reviewed remains consistently high in the health condition and body function and structure domains and low in the participation and personal factors domains. Additional focus also needs to be placed on categories of the ICF other than body function and structure in order to provide holistic care and planning for individuals with CP throughout all stages of their life. Comprehensive data will lead to a better understanding of the complex trajectories this heterogeneous and complicated condition presents with, and can be beneficial with planning for the future of newly diagnosed individuals.

Interesting findings were discovered in each of the CP Registry and Surveillance Groups Aims. Within Resource for CP Research, new ways research utilized CP registries/surveillance programs were identified. Extensive linking of multiple data sources in the Prevention aim resulted in integrated study data. In the Surveillance aim, traditional CP registries were not contributors of the highest number of articles on prevalence. Government datasets and collaborative CP registries were strong contributors to this aim, demonstrating successful alternative ways on gathering population-based data for CP research. The Planning aim addressed planning for current and future needs of children with CP and their families, and how communities can promote social and environmental changes for the success of this group. The poor representation of the Raise the Profile of Cerebral Palsy aim makes one question if there really is a serious lack of progress in this area or if traditional peer-reviewed journals are the best avenue for promotion and advocacy for CP. For example, international collaborations that include CP registries/surveillance programs have played a role in creating World CP Day [217].

While success of current CP registry/surveillance programs is commendable, adaptation to new technologies can improve access, reduce cost, and facilitate information transfer between registrants and registries, as well as between registries/surveillance programs. New and creative technology solutions can be adopted to reach participants on a regular basis, and facilitate researcher-health professional dialogue. In this era of social media and big data, we have the opportunity to improve the accuracy of information captured in real time, thus allowing for a better understanding of successful support strategies that maximize functioning, participation and well being for those with CP.

## Strengths and Limitations

The main strength of this study was performing a systematic search of several key literature databases using both standardized terminology and keywords (see Search Method for details). All steps of the review (i.e., article screening, data extraction, decision trees) were performed independently by two authors to minimize error. Further, the systematic review has been completed as per PRISMA guidelines. Finally, we focused on several factors that influence CP registry research (global region, CP registry/surveillance program classification, ICF domains, aims of the paper) with the goal of providing a thorough understanding of the relationship between CP registries/surveillance programs and the research they publish.

The main limitation to our study is that reference lists from the final 177 papers were not used as an additional resource for additional articles; therefore the results in this study might be under represented. Nevertheless, regions throughout the world were well represented in the search and a large number of publications were reviewed.

## Conclusion

CP registries/surveillance programs have made steady contributions of significant research discoveries over the past six years. Through the continued growth and collaboration between international CP registries/surveillance programs for research and epidemiological purposes, CP registries/surveillance programs can further impact surveillance and prevention of CP as well as promote public awareness, programming, and social change so as to improve life course outcomes for all individuals with cerebral palsy across the globe. Key stakeholders within CP research and healthcare delivery should encourage funding agencies to prioritize CP registries/surveillance programs as part of the agenda to accelerate discovery and care for this condition.

## Supplementary Material

S1

Supp

## Figures and Tables

**Table 1:** Articles Categorized by CP Registry classification and global region.

Registry classification	Europe	Australia	Canada	United States	Asia	Africa
Traditional CP registries	34	38	15	0	0	0
Regional collaborative CP registries	10	1	0	0	0	0
Subgroup of regional collaborative CP registries	14	0	0	0	0	0
CP surveillance programs	1	0	0	6	0	0
National health surveillance programs for CP	14	3	0	0	0	0
Government sponsored registries	27	2	1	4	1	0
Local surveillance groups	1	0	0	1	3	1
Total articles/region (% of 177 articles)	101 (58%)	44 (23%)	16 (9%)	11 (6%)	4 (2%)	1 (<1%)

**Table 2:** Registry classification and ICF domainsa represented.

Registry classification	HC	BSF	ACT	PART	ENV	PER
Traditional CP registries	18	47	21	1	19	6
Regional collaborative CP registries	8	3	2	0	1	0
Subgroup of regional collaborative CP registries	0	2	2	7	7	2
CP surveillance programs	3	1	2	0	1	1
National health surveillance programs for CP	1	12	6	1	3	1
Government sponsored registries	16	12	4	2	13	2
Local surveillance groups	5	1	1	0	0	0
Total for region (% of 177 articles)	51 (29%)	78 (43%)	38 (22%)	11 (6%)	44 (25%)	12 (7%)

HC: Health Condition; BSF: Body Structure and Function; ACT: Activities; PART: Participation; ENV: Environmental factors; PER: Personal Factors.

**Table 3:** Number of CP registries collecting variables related to ICF-CY code sets. This table lists the total number of ICF-CY codes [39,40] associated with each domain and age band. We evaluated the number of registries from the 14 surveys24 that addressed each code, and tallied according to domain and age band.

Age band		Body Structure and Function	Activities and Participation	Environmental Factors

0–2 years	Total codes	17	12	8
	All registries	4 (24%)	0 (0%)	0 (0%)
	Some registries	8 (47%)	6 (50%)	5 (63%)
	No registries	5 (29%)	6 (50%)	3 (37%)

3–5 years	Total codes	16	19	17
	All registries	2 (12%)	0 (0%)	4 (24%)
	Some registries	6 (38%)	10 (53%)	8 (47%)
	No registries	8 (50%)	9 (47%)	5 (29%)

6–12 years	Total codes	16	27	18
	All registries	0 (0%)	0 (0%)	0 (0%)
	Some registries	5 (31%)	2 (7%)	4 (22%)
	No registries	11 (69%)	25 (93%)	14 (88%)

13–17 years	Total codes	20	24	13
	All registries	2 (10%)	0 (0%)	0 (0%)
	Some registries	7 (35%)	1 (4%)	3 (23%)
	No registries	11 (55%)	23 (96%)	10 (77%)

## What this paper adds

- Seven different types of CP registries/surveillance programs were identified.
- Two new research uses for CP registries/surveillance programs were demonstrated.
- Recent articles addressed several ICF domains simultaneously.
- CP registries/surveillance programs are poorly equipped to research adolescent/adult issues.
- Alternative avenues must be used for Raising the Profile of CP.
