# Supplementary figures and images for: Systematic Review of Cerebral Palsy Registries/Surveillance Groups: Relationships between Registry Characteristics and Knowledge Dissemination
Source: Int J Phys Med Rehabil. Author manuscript; Available in PMC 2016 Oct 25. (PMC5079705; doi:10.4172/2329-9096.1000266)

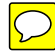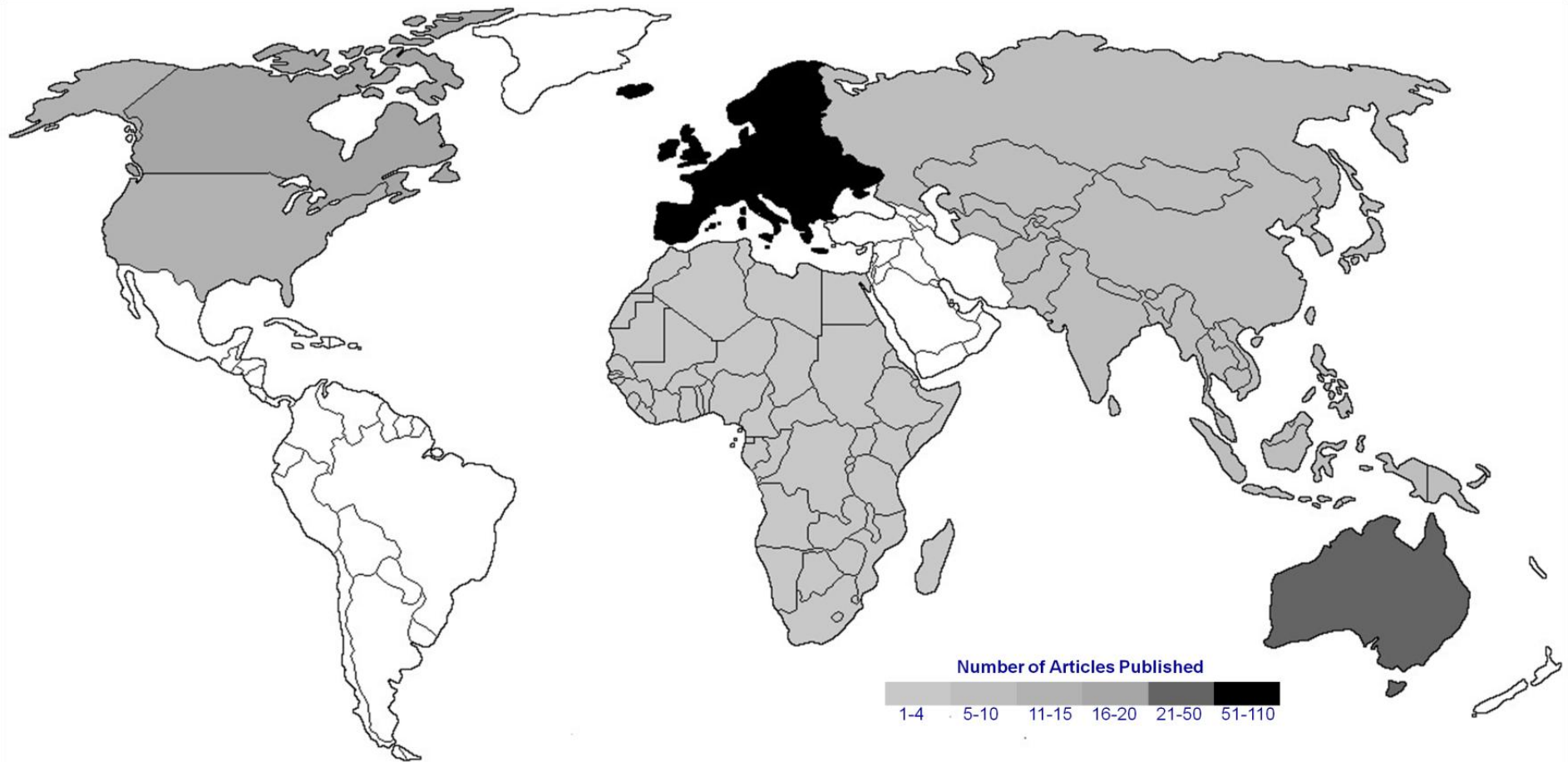

Supplement: S1 [file NIHMS816324-supplement-S1.pdf]
