# Supplementary material for: Systematic Review of Cerebral Palsy Registries/Surveillance Groups: Relationships between Registry Characteristics and Knowledge Dissemination
Source: Int J Phys Med Rehabil. Author manuscript; Available in PMC 2016 Oct 25. (PMC5079705; doi:10.4172/2329-9096.1000266)
Supplement: Supp [file NIHMS816324-supplement-Supp.docx]

**Table S1. Articles fulfilling *Resource for Cerebral Palsy Research* Aim**

| **Year** | **Article Title** | **Region^a^** | **Registry Type^b^** | **ICF Domains^c^** |
| --- | --- | --- | --- | --- |
| 2009 | Rasch measurement properties of the KIDSCREEN quality of life instrument in children with cerebral palsy and differential item functioning between children with and without cerebral palsy [^1^](#_ENREF_1) | EU | III | PER |
| 2009 | Participation in life situations of 8-12 year old children with cerebral palsy: cross sectional European study [^2^](#_ENREF_2) | EU | III | PART, ACT, ENV |
| 2009 | Characteristics influencing participation of Australian children with cerebral palsy [^3^](#_ENREF_3) | AU | I | PART |
| 2009 | Frequency of participation of 8-12-year-old children with cerebral palsy: a multi-centre cross-sectional European study [^4^](#_ENREF_4) | EU | III | PART |
| 2009 | The health of children with cerebral palsy and stress in their parents [^5^](#_ENREF_5) | EU | I | ENV, PER |
| 2009 | Magnetic resonance imaging findings in a population-based cohort of children with cerebral palsy [^6^](#_ENREF_6) | AU | I | BSF |
| 2009 | Upper limb orthoses and assistive technology utilization in children with hemiplegic cerebral palsy recruited from a population register [^7^](#_ENREF_7) | AU | I | ENV, PER |
| 2009 | Comorbidities in cerebral palsy and their relationship to neurologic subtype and GMFCS level [^8^](#_ENREF_8) | CA | I | BSF, ACT |
| 2009 | Motor impairments and activity limitations in children with spastic cerebral palsy: a Dutch population-based study [^9^](#_ENREF_9) | EU | IV | BSF, ACT |
| 2010 | Measuring the quality of life of children with cerebral palsy: comparing the conceptual differences and psychometric properties of three instruments [^10^](#_ENREF_10) | AU | I | ENV, PER |
| 2010 | Stability of caregiver-reported manual ability and gross motor function classifications of cerebral palsy [^11^](#_ENREF_11) | AU | I | ACT |
| 2010 | Cross-sectional comparison of periventricular leukomalacia in preterm and term children [^12^](#_ENREF_12) | CA | I | BSF |
| 2010 | Gestational age and basic school achievements: a national follow-up study in Denmark [^13^](#_ENREF_13) | EU | VI | PART |
| 2010 | Oromotor dysfunction and communication impairments in children with cerebral palsy: a register study [^14^](#_ENREF_14) | EU | I | BSF, ACT |
| 2010 | Pain in children with cerebral palsy: A cross-sectional multicentre European study [^15^](#_ENREF_15) | EU | III | BSF |
| 2010 | Sitting and standing performance in a total population of children with cerebral palsy: a cross-sectional study [^16^](#_ENREF_16) | EU | II | ACT |
| 2010 | Profile of refractive errors in cerebral palsy: impact of severity of motor impairment (GMFCS) and CP subtype on refractive outcome [^17^](#_ENREF_17) | EU | I | BSF |
| 2010 | A registry-based assessment of cerebral palsy and cerebral malformations [^18^](#_ENREF_18) | CA | I | BSF |
| 2010 | Behavioral and emotional symptoms of preschool children with cerebral palsy: a population-based study [^19^](#_ENREF_19) | EU | VI | PER |
| 2010 | Epilepsy in hemiplegic cerebral palsy due to perinatal arterial ischaemic stroke [^20^](#_ENREF_20) | AU | I | HC, ACT |
| 2010 | CP or not CP? A review of diagnoses in a cerebral palsy register [^21^](#_ENREF_21) | AU | I | HC |
| 2011 | Feasibility and reliability of classifying gross motor function among children with cerebral palsy using population-based record surveillance [^22^](#_ENREF_22) | US | IV | ACT |
| 2011 | Quantifying the physical, social and attitudinal environment of children with cerebral palsy [^23^](#_ENREF_23) | EU | III | PART, ENV, PER |
| 2011 | Unilateral cerebral palsy: a population-based study of gait and motor function [^24^](#_ENREF_24) | AU | I | BSF, ACT |
| 2011 | Prevalence of hip dislocation among children with cerebral palsy in regions with and without a surveillance programme: a cross sectional study in Sweden and Norway [^25^](#_ENREF_25) | EU | I | BSF |
| 2011 | Spasticity of the gastrosoleus muscle is related to the development of reduced passive dorsiflexion of the ankle in children with cerebral palsy: a registry analysis of 2,796 examinations in 355 children [^26^](#_ENREF_26) | EU | V | BSF |
| 2011 | Function and neuroimaging in cerebral palsy: a population-based study [^27^](#_ENREF_27) | EU | I | BSF, ACT |
| 2011 | Parenting stress and children with cerebral palsy: a European cross-sectional study [^28^](#_ENREF_28) | EU | III | ENV |
| 2011 | Classification of topographical pattern of spasticity in cerebral palsy: a registry perspective [^29^](#_ENREF_29) | AU | I | HC, BSF |
| 2011 | A population-based study and systematic review of hearing loss in children with cerebral palsy [^30^](#_ENREF_30) | AU | I | BSF |
| 2011 | Speech, expressive language and verbal cognition of preschool children with cerebral palsy in Iceland [^31^](#_ENREF_31) | EU | VI | BSF, ACT |
| 2011 | Population-based study of neuroimaging findings in children with cerebral palsy [^32^](#_ENREF_32) | CA | I | BSF |
| 2011 | Severe crouch gait in spastic diplegia can be prevented: a population-based study [^33^](#_ENREF_33) | AU | I | BSF, ACT |
| 2011 | Modified constraint induced therapy for children with hemiplegic cerebral palsy: randomized trial [^34^](#_ENREF_34) | AU | I | BSF, ACT |
| 2012 | Predictors of drop-out in a multi-centre longitudinal study of participation and quality of life of children with cerebral palsy [^35^](#_ENREF_35) | EU | III | ENV |
| 2012 | Botulinum neurotoxin treatment in children with cerebral palsy: a population-based study in Norway [^36^](#_ENREF_36) | EU | I | BSF |
| 2012 | Low 5-min Apgar score in moderately preterm infants; association with subsequent death and cerebral palsy: a register based Danish national study [^37^](#_ENREF_37) | EU | VI | HC |
| 2012 | Characteristics of children with cerebral palsy in the ORACLE children study [^38^](#_ENREF_38) | EU | II | BSF, HC |
| 2012 | Passive range of motion in a population-based sample of children with spastic cerebral palsy who walk [^39^](#_ENREF_39) | EU | I | BSF. ACT |
| 2012 | Recruitment bias and characteristics of participants with severe cerebral palsy in a cross-sectional survey [^40^](#_ENREF_40) | EU | I | PER |
| 2012 | Reliability of GMFCS family report questionnaire [^41^](#_ENREF_41) | EU | I | ACT |
| 2012 | Better walking performance in older children with cerebral palsy [^42^](#_ENREF_42) | EU | I | ACT |
| 2012 | Congenital non-central nervous system malformations in cerebral palsy: a distinct subset? [^43^](#_ENREF_43) | CA | I | BSF |
| 2013 | The use of bioelectrical impedance analysis to estimate total body water in young children with cerebral palsy [^44^](#_ENREF_44) | AU | I | BSF |
| 2013 | Normal imaging in patients with cerebral palsy: what does it tell us? [^45^](#_ENREF_45) | CA | I | BSF |
| 2013 | Description and psychometric properties of the CP QOL-Teen: A quality of life questionnaire for adolescents with cerebral palsy [^46^](#_ENREF_46) | AU | I | ENV, PER |
| 2013 | The agreement between GMFCS and GMFCS-E&R in children with cerebral palsy [^47^](#_ENREF_47) | EU | V | ACT |
| 2013 | Children with cerebral palsy and periventricular white matter injury: does gestational age affect functional outcome? [^48^](#_ENREF_48) | AU | I | BSF, ACT |
| 2013 | Communication ability in cerebral palsy: a study from the CP register of western Sweden [^49^](#_ENREF_49) | EU | I | ACT |
| 2013 | Term neonatal encephalopathy antecedent cerebral palsy: a retrospective population-based study [^50^](#_ENREF_50) | CA | I | BSF, ACT |
| 2013 | Physical activity in a total population of children and adolescents with cerebral palsy [^51^](#_ENREF_51) | EU | V | PART |
| 2013 | Apolipoprotein E polymorphisms and severity of cerebral palsy: a cross-sectional study in 255 children in Norway [^52^](#_ENREF_52) | EU | I | BSF |
| 2013 | Brain injury in very preterm children and neurosensory and cognitive disabilities during childhood: the EPIPAGE cohort study [^53^](#_ENREF_53) | EU | VI | BSF |
| 2013 | The relationship between gross motor function and manual ability in cerebral palsy [^54^](#_ENREF_54) | CA | I | ACT |
| 2013 | Pain in young people aged 13 to 17 years with cerebral palsy: cross-sectional, multicentre European study [^55^](#_ENREF_55) | EU | III | BSF |
| 2013 | Reliable classification of functional profiles and movement disorders of children with cerebral palsy [^56^](#_ENREF_56) | AU | I | BSF, ACT |
| 2013 | Postural asymmetries in young adults with cerebral palsy [^57^](#_ENREF_57) | EU | V | BSF, ACT |
| 2013 | Item response theory and structural equation modeling for ordinal data: Describing the relationship between KIDSCREEN and Life-H [^58^](#_ENREF_58) | EU | III | PART, PER |
| 2014 | Spectrum of visual disorders in a population-based cerebral palsy cohort [^59^](#_ENREF_59) | CA | I | BSF |
| 2014 | How are actual needs recognized in the content and goals of written rehabilitation plans? [^60^](#_ENREF_60) | EU | VI | ENV |
| 2014 | Gene sequences regulating the production of apoE and cerebral palsy of variable severity [^61^](#_ENREF_61) | EU | I | BSF |
| 2014 | European study of frequency of participation of adolescents with and without cerebral palsy [^62^](#_ENREF_62) | EU | III | PART |
| 2014 | Stability of motor function and associated impairments between childhood and adolescence in young people with cerebral palsy in Europe [^63^](#_ENREF_63) | EU | III | ACT |

^a^AF = Africa; AS = Asia AU = Australia; CA = Canada; EU = Europe; US = United States of America

^b^I = traditional CP registries; II =regional collaborative registries ; III = subgroups of regional collaborative registries ; IV = CP surveillance programs; V = government sponsored CP health programs; VI = government sponsored registries; VII = local surveillance programs.

^c^HC = health condition; BSF = body structures and function; ACT = activities; PART = participation; ENV = environmental factors; PER = personal factors.

**Table S2. Articles fulfilling *Surveillance* Aim**

| **Year** | **Article Title** | **Region** | **Registry Type** | **ICF Domains** |
| --- | --- | --- | --- | --- |
| 2009 | Prevalence of cerebral palsy: Autism and Developmental Disabilities Monitoring Network, three sites, United States, 2004 [^64^](#_ENREF_64) | US | IV | HC |
| 2009 | Neurological disorders in children and adolescents [^65^](#_ENREF_65) | AS | VII | HC |
| 2009 | Trends in prevalence and characteristics of cerebral palsy among Icelandic children born 1990 to 2003 [^66^](#_ENREF_66) | EU | VI | HC |
| 2009 | Children with cerebral palsy: severity and trends over time [^67^](#_ENREF_67) | EU | II | HC |
| 2010 | Door-to-door survey of major neurological disorders in Al Kharga District, New Valley, Egypt: methodological aspects [^68^](#_ENREF_68) | AF | VII | HC |
| 2010 | The changing panorama of cerebral palsy in Sweden. X. Prevalence and origin in the birth-year period 1999-2002 [^69^](#_ENREF_69) | EU | I | HC |
| 2010 | Cerebral palsy among term and postterm births [^70^](#_ENREF_70) | EU | VI | HC |
| 2010 | Cerebral palsy in eastern Denmark: declining birth prevalence but increasing numbers of unilateral cerebral palsy in birth year period 1986-1998 [^71^](#_ENREF_71) | EU | I | HC |
| 2010 | Trends in prevalence of cerebral palsy in children born with a birthweight of 2,500 g or over in Europe from 1980 to 1998 [^72^](#_ENREF_72) | EU | II | HC |
| 2011 | Cerebral palsy among children born moderately preterm or at moderately low birthweight between 1980 and 1998: A European register-based study [^73^](#_ENREF_73) | EU | II | HC |
| 2011 | Cerebral palsy rates by birth weight, gestation and severity in North of England, 1991-2000 singleton births [^74^](#_ENREF_74) | EU | I | HC, BSF |
| 2011 | Prevalence and functioning of children with cerebral palsy in four areas of the United States in 2006: a report from the Autism and Developmental Disabilities Monitoring Network [^75^](#_ENREF_75) | US | IV | HC |
| 2011 | Prevalence of cerebral palsy in children < 10 years of age in R.S. Pura town of Jammu and Kashmir [^76^](#_ENREF_76) | AS | VII | HC |
| 2011 | Distribution of motor types in cerebral palsy: how do registry data compare? [^77^](#_ENREF_77) | AU | I | HC, BSF |
| 2011 | Rates of cerebral palsy in Victoria, Australia, 1970 to 2004: has there been a change? [^78^](#_ENREF_78) | AU | I | HC |
| 2012 | Survival and neurodevelopmental outcome of ELBW children at 5 years of age: comparison of two cohorts born 10 years apart [^79^](#_ENREF_79) | EU | VI | HC |
| 2012 | High prevalence of major neurological disorders in two Albanian communities: results of a door-to-door survey [^80^](#_ENREF_80) | EU | VII | HC |
| 2012 | Scoliosis in a total population of children with cerebral palsy [^81^](#_ENREF_81) | EU | V | BSF |
| 2012 | Survival of individuals with cerebral palsy born in Victoria, Australia, between 1970 and 2004 [^82^](#_ENREF_82) | AU | I | HC |
| 2012 | Epilepsy and cerebral palsy: characteristics and trends in children born in 1976-1998 [^83^](#_ENREF_83) | EU | II | HC |
| 2012 | Autism spectrum disorder, ADHD, epilepsy, and cerebral palsy in Norwegian children [^84^](#_ENREF_84) | EU | VI | HC |
| 2012 | Cerebral palsy registers and high-quality data: an evaluation of completeness of the 4Child register using capture-recapture techniques [^85^](#_ENREF_85) | EU | II | HC |
| 2013 | Sex differences in cerebral palsy incidence and functional ability: a total population study [^86^](#_ENREF_86) | EU | I | ACT |
| 2013 | Trends in prevalence and characteristics of post-neonatal cerebral palsy cases: a European registry-based study [^87^](#_ENREF_87) | EU | II | HC |
| 2013 | Prevalence of early childhood disability in a rural district of Sind, Pakistan [^88^](#_ENREF_88) | AS | VII | HC |
| 2013 | Prevalence of cerebral palsy in Quebec: alternative approaches [^89^](#_ENREF_89) | CA | I | HC |
| 2013 | Differences across counties in the registered prevalence of autism, ADHD, epilepsy and cerebral palsy in Norway [^90^](#_ENREF_90) | EU | VI | HC |
| 2013 | Follow-up study of 2-year-olds born at very low gestational age in Estonia [^91^](#_ENREF_91) | EU | VI | HC, BSF |
| 2013 | The role of migration and choice of denominator on the prevalence of cerebral palsy [^92^](#_ENREF_92) | US | IV | HC, ENV |
| 2014 | Prevalence of cerebral palsy, co-occurring autism spectrum disorders, and motor functioning - Autism and Developmental Disabilities Monitoring Network, USA, 2008 [^93^](#_ENREF_93) | US | IV | HC |
| 2014 | The panorama of cerebral palsy in Sweden. XI. Changing patterns in the birth-year period 2003-2006 [^94^](#_ENREF_94) | EU | I | HC |
| 2014 | Trends in the prevalence of cerebral palsy among very preterm infants (<31 weeks' gestational age) [^95^](#_ENREF_95) | CA | VI | HC |

^a^AF = Africa; AS = Asia AU = Australia; CA = Canada; EU = Europe; US = United States of America

^b^I = traditional CP registries; II =regional collaborative registries ; III = subgroups of regional collaborative registries ; IV = CP surveillance programs; V = government sponsored CP health programs; VI = government sponsored registries; VII = local surveillance programs.

^c^HC = health condition; BSF = body structures and function; ACT = activities; PART = participation; ENV = environmental factors; PER = personal factors.

**Table S3. Articles fulfilling *Surveillance + Resource for CP Research* Aims**

| **Year** | **Article Title** | **Region** | **Registry Type** | **ICF Domains** |
| --- | --- | --- | --- | --- |
| 2009 | Dyskinetic cerebral palsy in Europe: trends in prevalence and severity [^96^](#_ENREF_96) | EU | II | HC, BSF, ACT |
| 2009 | Motor function in 5-year-old children with cerebral palsy in the South Australian population [^97^](#_ENREF_97) | AU | I | ACT |
| 2010 | Socio-economic inequalities in cerebral palsy prevalence in the United Kingdom: a register-based study [^98^](#_ENREF_98) | EU | II | ENV |
| 2010 | Congenital anomalies in children with cerebral palsy: a population-based record linkage study [^99^](#_ENREF_99) | EU | I | HC, BSF |
| 2011 | Using the Gross Motor Function Classification System to describe patterns of motor severity in cerebral palsy [^100^](#_ENREF_100) | AU | I | HC, ACT |
| 2012 | Children with cerebral palsy: racial disparities in functional limitations [^101^](#_ENREF_101) | US | IV | PER |

^a^AF = Africa; AS = Asia AU = Australia; CA = Canada; EU = Europe; US = United States of America

^b^I = traditional CP registries; II =regional collaborative registries ; III = subgroups of regional collaborative registries ; IV = CP surveillance programs; V = government sponsored CP health programs; VI = government sponsored registries; VII = local surveillance programs.

^c^HC = health condition; BSF = body structures and function; ACT = activities; PART = participation; ENV = environmental factors; PER = personal factors.

**Table S4. Articles fulfilling *Prevention* Aim**

| **Year** | **Article Title** | **Region** | **Registry Type** | **ICF Domains** |
| --- | --- | --- | --- | --- |
| 2009 | Prenatal stress and cerebral palsy: a nationwide cohort study in Denmark [^102^](#_ENREF_102) | EU | VI | ENV |
| 2010 | Parental infertility and cerebral palsy in children [^103^](#_ENREF_103) | EU | I | ENV |
| 2012 | Antenatal factors associated with perinatal arterial ischemic stroke [^104^](#_ENREF_104) | EU | VI | BSF, ENV |
| 2012 | Reproductive technologies and the risk of birth defects [^105^](#_ENREF_105) | AU | VI | ENV |
| 2012 | Does antenatal tobacco or alcohol exposure influence a child's cerebral palsy? A population-based study [^106^](#_ENREF_106) | CA | I | ENV |
| 2012 | Risk factors associated with cerebral palsy in preterm infants [^107^](#_ENREF_107) | US | VI | BSF |
| 2013 | Cerebral palsy and perinatal infection in children born at term [^108^](#_ENREF_108) | EU | I | ENV |
| 2013 | Non-infectious risk factors for different types of cerebral palsy in term-born babies: a population-based, case-control study [^109^](#_ENREF_109) | EU | I | BSF, ENV |
| 2013 | Maternal diagnosis of obesity and risk of cerebral palsy in the child [^110^](#_ENREF_110) | US | VI | ENV |
| 2013 | Neonatal infection and 5-year neurodevelopmental outcome of very preterm infants [^111^](#_ENREF_111) | EU | VI | ENV |
| 2014 | Risk of cerebral palsy in relation to pregnancy disorders and preterm birth: a national cohort study [^112^](#_ENREF_112) | EU | VI | ENV |
| 2014 | "Chorioamnionitis and cerebral palsy: Lessons from a patient registry [^113^](#_ENREF_113) | CA | I | BSF |

^a^AF = Africa; AS = Asia AU = Australia; CA = Canada; EU = Europe; US = United States of America

^b^I = traditional CP registries; II =regional collaborative registries ; III = subgroups of regional collaborative registries ; IV = CP surveillance programs; V = government sponsored CP health programs; VI = government sponsored registries; VII = local surveillance programs.

^c^HC = health condition; BSF = body structures and function; ACT = activities; PART = participation; ENV = environmental factors; PER = personal factors.

**Table S5. Articles fulfilling *Planning + Prevention* Aims**

| **Year** | **Article Title** | **Region** | **Registry Type** | **ICF Domains** |
| --- | --- | --- | --- | --- |
| 2009 | Predictors of cerebral palsy in very preterm infants: the EPIPAGE prospective population-based cohort study [^114^](#_ENREF_114) | EU | VI | HC, BSF |
| 2010 | Predicting neurosensory disabilities at two years of age in a national cohort of extremely premature infants [^115^](#_ENREF_115) | EU | VI | ENV, BSF |

^a^AF = Africa; AS = Asia AU = Australia; CA = Canada; EU = Europe; US = United States of America

^b^I = traditional CP registries; II =regional collaborative registries ; III = subgroups of regional collaborative registries ; IV = CP surveillance programs; V = government sponsored CP health programs; VI = government sponsored registries; VII = local surveillance programs.

^c^HC = health condition; BSF = body structures and function; ACT = activities; PART = participation; ENV = environmental factors; PER = personal factors.

**Table S6. Articles fulfilling *Prevention + Resource for CP Research* Aims**

| **Year** | **Article Title** | **Region** | **Registry Type** | **ICF Domains** |
| --- | --- | --- | --- | --- |
| 2009 | Genetic susceptibility to viral exposure may increase the risk of cerebral palsy [^116^](#_ENREF_116) | AU | I | BSF |
| 2009 | The risk of cerebral palsy in survivors of multiple pregnancies with cofetal loss or death [^117^](#_ENREF_117) | AU | I | ENV |
| 2009 | Short and long-term effects of antenatal corticosteroids assessed in a cohort of 7,827 children born preterm [^118^](#_ENREF_118) | EU | VI | HC |
| 2009 | Is breech presentation a risk factor for cerebral palsy? A Norwegian birth cohort study [^119^](#_ENREF_119) | EU | I | BSF |
| 2010 | Adverse obstetric events are associated with significant risk of cerebral palsy [^120^](#_ENREF_120) | US | VI | ENV, HC |
| 2010 | Multiplicity and early gestational age contribute to an increased risk of cerebral palsy from assisted conception: a population-based cohort study [^121^](#_ENREF_121) | EU | VI | HC, ENV |
| 2010 | Cerebral palsy in children born after in vitro fertilization. Is the risk decreasing? [^122^](#_ENREF_122) | EU | VI | HC, ENV |
| 2010 | Association of cerebral palsy with Apgar score in low and normal birthweight infants: population based cohort study [^123^](#_ENREF_123) | EU | I | BSF |
| 2010 | Cerebral palsy and assisted reproductive technologies: a case-control study [^124^](#_ENREF_124) | AU | I | BSF |
| 2011 | Migraine and preterm birth [^125^](#_ENREF_125) | AU | I | ENV, HC |
| 2011 | Placental infarction identified by macroscopic examination and risk of cerebral palsy in infants at 35 weeks of gestational age and over [^126^](#_ENREF_126) | AU | I | BSF |
| 2011 | Epidemiologic associations with cerebral palsy [^127^](#_ENREF_127) | AU | I | ENV |
| 2011 | Induction of labor and cerebral palsy: a population-based study in Norway [^128^](#_ENREF_128) | EU | I | ENV |
| 2011 | Population case-control study of cerebral palsy: neonatal predictors for low-risk term singletons [^129^](#_ENREF_129) | AU | I | BSF |
| 2011 | Ethnic and socioeconomic disparities in prevalence of cerebral palsy [^130^](#_ENREF_130) | US | VI | PER |
| 2012 | Heavy maternal alcohol consumption and cerebral palsy in the offspring [^131^](#_ENREF_131) | AU | I | ENV |
| 2012 | Fetal and Maternal Candidate Single Nucleotide Polymorphism Associations With Cerebral Palsy: A Case-Control Study [^132^](#_ENREF_132) | AU | I | BSF |
| 2012 | Cerebral palsy and neonatal death in term singletons born small for gestational age [^133^](#_ENREF_133) | EU | I | HC |
| 2012 | The effects of multiple pre- and perinatal risk factors on the occurrence of cerebral palsy. A Norwegian register based study [^134^](#_ENREF_134) | EU | I | BSF |
| 2013 | Antecedents of cerebral palsy and perinatal death in term and late preterm singletons [^135^](#_ENREF_135) | AU | I | ENV, BSF |
| 2013 | Maternal infections during pregnancy and cerebral palsy: a population-based cohort study [^136^](#_ENREF_136) | EU | I | ENV |
| 2013 | Mediators of the association between pre-eclampsia and cerebral palsy: population based cohort study [^137^](#_ENREF_137) | EU | I | HC, ENV |
| 2013 | Genetic and clinical contributions to cerebral palsy: a multi-variable analysis [^138^](#_ENREF_138) | AU | I | BSF |
| 2013 | Risk of cerebral palsy and childhood epilepsy related to infections before or during pregnancy [^139^](#_ENREF_139) | EU | VI | HC |
| 2014 | Risk of cerebral palsy in term-born singletons according to growth status at birth [^140^](#_ENREF_140) | EU | I | BSF |
| 2014 | An Australian population study of factors associated with MRI patterns in cerebral palsy [^141^](#_ENREF_141) | AU | I | BSF |
| 2014 | Congenital cytomegalovirus is associated with severe forms of cerebral palsy and female sex in a retrospective population-based study [^142^](#_ENREF_142) | AU | II | BSF, ACT |

^a^AF = Africa; AS = Asia AU = Australia; CA = Canada; EU = Europe; US = United States of America

^b^I = traditional CP registries; II =regional collaborative registries ; III = subgroups of regional collaborative registries ; IV = CP surveillance programs; V = government sponsored CP health programs; VI = government sponsored registries; VII = local surveillance programs.

^c^HC = health condition; BSF = body structures and function; ACT = activities; PART = participation; ENV = environmental factors; PER = personal factors.

**Table S7. Articles fulfilling *Planning* Aim**

| **Year** | **Article Title** | **Region** | **Registry Type** | **ICF Domains** |
| --- | --- | --- | --- | --- |
| 2009 | Morbidities and hospital resource use during the first 3 years of life among very preterm infants [^143^](#_ENREF_143) | EU | VI | HC, BSF |
| 2009 | Lifetime costs of cerebral palsy [^144^](#_ENREF_144) | EU | I | ENV |
| 2009 | Socio-economic achievements of individuals born very preterm at the age of 27 to 29 years: a nationwide cohort study [^145^](#_ENREF_145) | EU | VI | PART |
| 2010 | Impact of very preterm birth on health care costs at five years of age [^146^](#_ENREF_146) | EU | VI | HC, ENV |
| 2011 | Prediction of neurodevelopmental and sensory outcome at 5 years in Norwegian children born extremely preterm [^147^](#_ENREF_147) | EU | VI | HC, BSF, ACT |
| 2012 | Prediction of outcome at 5 years from assessments at 2 years among extremely preterm children: A Norwegian national cohort study [^148^](#_ENREF_148) | EU | VI | HC, BSF, ACT |

^a^AF = Africa; AS = Asia AU = Australia; CA = Canada; EU = Europe; US = United States of America

^b^I = traditional CP registries; II =regional collaborative registries ; III = subgroups of regional collaborative registries ; IV = CP surveillance programs; V = government sponsored CP health programs; VI = government sponsored registries; VII = local surveillance programs.

^c^HC = health condition; BSF = body structures and function; ACT = activities; PART = participation; ENV = environmental factors; PER = personal factors.

**Table S8. Articles fulfilling *Planning + Resource for CP Research* Aims**

| **Year** | **Article Title** | **Region** | **Registry Type** | **ICF Domains** |
| --- | --- | --- | --- | --- |
| 2009 | Development of lower limb range of motion from early childhood to adolescence in cerebral palsy: a population-based study [^149^](#_ENREF_149) | EU | V | BSF |
| 2009 | The relationship of cerebral palsy subtype and functional motor impairment: a population-based study [^150^](#_ENREF_150) | CA | I | HC, ACT |
| 2010 | Use of manual and powered wheelchair in children with cerebral palsy: a cross-sectional study [^151^](#_ENREF_151) | EU | V | ENV, ACT |
| 2010 | Determinants of ambulation in children with spastic quadriplegic cerebral palsy: a population-based study [^152^](#_ENREF_152) | CA | I | BSF, ACT |
| 2011 | Access of children with cerebral palsy to the physical, social and attitudinal environment they need: a cross-sectional European study [^153^](#_ENREF_153) | EU | III | PART, ENV, PER |
| 2011 | Predicting comorbidities with neuroimaging in children with cerebral palsy [^154^](#_ENREF_154) | CA | I | BSF |
| 2011 | Should children with cerebral palsy and normal imaging undergo testing for inherited metabolic disorders? [^155^](#_ENREF_155) | AU | I | BSF |
| 2012 | Association between participation in life situations of children with cerebral palsy and their physical, social, and attitudinal environment: a cross-sectional multicenter European study [^156^](#_ENREF_156) | EU | III | PART, ENV, PER |
| 2012 | Feeding problems, growth and nutritional status in children with cerebral palsy [^157^](#_ENREF_157) | EU | I | BSF |
| 2012 | Gastrostomy tube feeding of children with cerebral palsy: variation across six European countries [^158^](#_ENREF_158) | EU | III | BSF |
| 2012 | Unilateral varus osteotomy of the proximal femur in children with cerebral palsy: a five-year follow-up of the development of both hips [^159^](#_ENREF_159) | EU | II | BSF |
| 2012 | Prevalence and predictors of drooling in 7- to 14-year-old children with cerebral palsy: a population study [^160^](#_ENREF_160) | AU | I | BSF |
| 2012 | The natural history of hip development in cerebral palsy [^161^](#_ENREF_161) | EU | V | BSF |
| 2013 | Immunisation coverage in children with cerebral palsy compared with the general population [^162^](#_ENREF_162) | AU | I | ENV |
| 2013 | Social outcomes of young adults with cerebral palsy [^163^](#_ENREF_163) | AU | I | ACT, ENV |
| 2013 | Determinants of inclusive education of 8-12 year old children with cerebral palsy in 9 European regions [^164^](#_ENREF_164) | EU | III | ENV |
| 2013 | Neurodevelopmental outcome in extremely preterm infants at 2.5 years after active perinatal care in Sweden [^165^](#_ENREF_165) | EU | VI | BSF, ACT |
| 2013 | Congenital cerebral palsy, child sex and parent cardiovascular risk [^166^](#_ENREF_166) | EU | I | ENV |
| 2013 | Fractures in children with cerebral palsy: a total population study [^167^](#_ENREF_167) | EU | V | HC, BSF, ACT, PER, ENV |
| 2014 | Outpatient physical therapy utilization for children and adolescents with intellectual disabilities in Taiwan: a population-based nationwide study [^168^](#_ENREF_168) | AS | VI | ENV |
| 2014 | Hip displacement in relation to age and gross motor function in children with cerebral palsy [^169^](#_ENREF_169) | EU | V | BSF |

^a^AF = Africa; AS = Asia AU = Australia; CA = Canada; EU = Europe; US = United States of America

^b^I = traditional CP registries; II =regional collaborative registries ; III = subgroups of regional collaborative registries ; IV = CP surveillance programs; V = government sponsored CP health programs; VI = government sponsored registries; VII = local surveillance programs.

^c^HC = health condition; BSF = body structures and function; ACT = activities; PART = participation; ENV = environmental factors; PER = personal factors.

**Table S9. Articles fulfilling *Planning + Surveillance* Aims**

| **Year** | **Article Title** | **Region** | **Registry Type** | **ICF Domains** |
| --- | --- | --- | --- | --- |
| 2011 | Survival at 19 years of age in a total population of children and young people with cerebral palsy [^170^](#_ENREF_170) | EU | V | ENV |

^a^AF = Africa; AS = Asia AU = Australia; CA = Canada; EU = Europe; US = United States of America

^b^I = traditional CP registries; II =regional collaborative registries ; III = subgroups of regional collaborative registries ; IV = CP surveillance programs; V = government sponsored CP health programs; VI = government sponsored registries; VII = local surveillance programs.

^c^HC = health condition; BSF = body structures and function; ACT = activities; PART = participation; ENV = environmental factors; PER = personal factors.

**Table S10. Articles fulfilling *Raising the profile of CP + Resource for CP Research* Aims**

| **Year** | **Article Title** | **Region** | **Registry Type** | **ICF Domains** |
| --- | --- | --- | --- | --- |
| 2009 | Hip surveillance in Tasmanian children with cerebral palsy [^171^](#_ENREF_171) | AU | VI | BSF |
| 2009 | A classification system for hip disease in cerebral palsy [^172^](#_ENREF_172) | AU | V | BSF |
| 2010 | To what extent do children with cerebral palsy participate in everyday life situations? [^173^](#_ENREF_173) | EU | I | PART |
| 2011 | The cerebral palsy research registry: development and progress toward national collaboration in the United States [^174^](#_ENREF_174) | US | VII | BSF, ACT |
| 2011 | Five-year outcome of state-wide hip surveillance of children and adolescents with cerebral palsy [^175^](#_ENREF_175) | AU | V | BSF |
| 2011 | The Consensus Statement on Hip Surveillance for Children with Cerebral Palsy: Australian Standards of Care [^176^](#_ENREF_176) | AU | V | BSF |

^a^AF = Africa; AS = Asia AU = Australia; CA = Canada; EU = Europe; US = United States of America

^b^I = traditional CP registries; II =regional collaborative registries ; III = subgroups of regional collaborative registries ; IV = CP surveillance programs; V = government sponsored CP health programs; VI = government sponsored registries; VII = local surveillance programs.

^c^HC = health condition; BSF = body structures and function; ACT = activities; PART = participation; ENV = environmental factors; PER = personal factors.

**Table S11. Articles fulfilling *Raising the profile of CP* Aim**

| **Year** | **Article Title** | **Region** | **Registry Type** | **ICF Domains** |
| --- | --- | --- | --- | --- |
| 2013 | Collaborating with consumers: the key to achieving statutory notification for birth defects and cerebral palsy in Western Australia [^177^](#_ENREF_177) | AU | I | PER |

^a^AF = Africa; AS = Asia AU = Australia; CA = Canada; EU = Europe; US = United States of America

^b^I = traditional CP registries; II =regional collaborative registries ; III = subgroups of regional collaborative registries ; IV = CP surveillance programs; V = government sponsored CP health programs; VI = government sponsored registries; VII = local surveillance programs.

^c^HC = health condition; BSF = body structures and function; ACT = activities; PART = participation; ENV = environmental factors; PER = personal factors.

**Table S12. CP registries found in reviewed articles, organized by type and region**

| **Registry type^a^** | I | II | III | IV | V | VI | VII |
| --- | --- | --- | --- | --- | --- | --- | --- |
| **Europe^b^**  (n=26) | 1 CPRWS*  2 DNCPR  3 NCPR*  4 NECCPS*  5 NICPR  6 REHOP* | 1 4Child  2 SCPE*  3 UKCP | 1 SPARCLE | 1 GCPS | 1 CPOP  2 CPUP* | 1 DNPR  2 ENNRR  3 EPIPAGE  4 EXPRESS  5 FHDR  6 FSII  7 ISDCC  8 NMBR  9 NNII  10 NPR  11 PMSI  12 SPR | 1 HSTS |
| **Australia^c^**  (n=7) | 1 SACPR  2 VCPR*  3 WACPR* | 1 ACPR* | -- | -- | 1 QHS  2 THS  3 VHS | 1 SABDR | -- |
| **Canada^d^**  (n=2) | 1 REPACQ | -- | -- | -- | -- | 1 NSPFUP | -- |
| **United States^e^**  (n=4) | -- | -- | -- | 1 ADDMN*  2 MADDSP* | -- | 1 CDDS | 1 CPRR* |
| **Asia^f^**  (n=4) | -- | -- | -- | -- | -- | 1 TNHI | 1 HSK  2 HSRSP  3 HSTR |
| **Africa^g^**  (n=1) | -- | -- | -- | -- | -- | -- | 1 ENDAKD |
| **Total registry of type** | 11 | 4 | 1 | 3 | 5 | 16 | 6 |

^a^I = traditional CP registries; II =regional collaborative registries ; III = subgroups of regional collaborative registries ; IV = CP surveillance programs; V = government sponsored CP health programs; VI = government sponsored registries; VII = local surveillance programs.

^b^CPRWS = CP Register of Western Sweden; DNCPR = Danish National CP Registry; NCPR = Norwegian CP Register; NECCPS = North of England Collaborative CP Survey; NICPR = Northern Ireland Cerebral Palsy Register; REHOP = Registre des Handicaps de L’Enfant et Observatoire Périnatal [France]; 4Child = Four Counties Database of Cerebral Palsy, Vision Loss and Hearing Loss in Children; SCPE = Surveillance of CP in Europe; UKCPR = United Kingdom CP collaborative network; SPARCLE = Study of PARticipation of Children with CP Living in Europe; GCPS = Gelderland CP Survey [Netherlands]; CPOP = CP Oppfølgings Program; CPUP = CP Uppföljnings Program; DNPR = Danish National Patients Registry; ENNRR = Estonia National Neonatal Research Register; EPIPAGE = Etude EPIdémiologique sur les Petits Ages GEstationnels [France]; EXPRESS = Extremely Preterm Infants Study in Sweden; FHDR = Finland Hospital Discharge Register; FSII = Finland Social Insurance Institution; ISDCC = Iceland State Diagnostic and Counselling Centre; NMBR = Norwegian Medical Birth Registry; NNII = Norwegian National Insurance Scheme; NPR = Norwegian Patient Register; PMSI = Programme de Médicalisation des Systèmes d'Information [France]; SPR = Swedish Patient Register [formerly called Hospital Discharge Record]; HSTS = Household-survey in Tirana and Saranda [Albania].

^c^SACPR = Southern Australia CP Registry; VCPR = Victorian CP Register; WACPR = Western Australia CP Register; ACPR = Australian CP Register; QHS = Queensland Hip Surveillance; THS = Tasmania Hip Surveillance; VHS = Victorian Hip Surveillance; SABDR = South Australian Birth Defects Register

^d^REPACQ = Registre de la paralysie cérébrale au Québec ; NSPFUP = Nova Scotia Perinatal Follow-Up Program.

^e^ADDMN = Autism and Developmental Disabilities Monitoring Network; MADDSP = Metropolitan Atlanta Developmental Disabilities Surveillance Program; CDDS = California Department of Developmental Services Database; CPRR = CP Research Registry.

^f^TNHI = Taiwan National Health Insurance; HSK = Household-survey in Kolkata [India]; HSRSP = Household-survey in Ranbir Singh Pura [India]; HSTR = Household-survey in Taluka-Rohr [Pakistan]

^g^ENDAKD = Epidemiology of Neurological Disorders in Al Kharga District [Egypt].

*Data elements available for ICF-CY review (see Table 3, text for further details).**Table S13. Aims addressed in papers by each registry type**

|  | **Registry type^a^** | | | | | | |
| --- | --- | --- | --- | --- | --- | --- | --- |
| **Aim** **addressed** | **I** | **II** | **III** | **IV** | **V** | **VI** | **VII** |
| *Planning* | 1 | 0 | 0 | 0 | 5 | 0 | 0 |
| *Planning + Prevention* | 0 | 0 | 0 | 0 | 0 | 2 | 0 |
| *Planning + Resource* | 10 | 1 | 3 | 5 | 0 | 2 | 0 |
| *Planning + Surveillance* | 0 | 0 | 0 | 0 | 1 | 0 | 0 |
| *Prevention* | 5 | 0 | 0 | 0 | 0 | 7 | 0 |
| *Prevention + Resource for CP Research* | 20 | 1 | 0 | 1 | 0 | 5 | 0 |
| *Raising the Profile of CP* | 1 | 0 | 0 | 0 | 0 | 0 | 0 |
| *Raising the Profile of CP + Resource for CP Research* | 1 | 0 | 0 | 0 | 4 | 0 | 1 |
| *Resource for CP Research* | 37 | 1 | 11 | 2 | 6 | 6 | 0 |
| *Resource for CP Research + Surveillance* | 3 | 2 | 0 | 1 | 0 | 0 | 0 |
| *Surveillance* | 9 | 5 | 0 | 4 | 2 | 7 | 5 |
| **TOTAL** | 87 | 10 | 14 | 13 | 18 | 29 | 6 |

^a^I = traditional CP registries; II =regional collaborative registries ; III = subgroups of regional collaborative registries ; IV = CP surveillance programs; V = government sponsored CP health programs; VI = government sponsored registries; VII = local surveillance programs.
